# The value of non-contrast chest CT in the prediction of myocardial injury in patients with the COVID-19 Omicron variant

**DOI:** 10.1038/s41598-023-37335-2

**Published:** 2023-06-26

**Authors:** Ying Zhong, Zhenggang Sun, Ping Xu, Yun Bai, Zheng Zhang, Guan Wang

**Affiliations:** Department of Radiology, The First Hospital of China Medical University, No. 155, North Nanjing Street, Shenyang, 110001 Liaoning China

**Keywords:** Cardiology, Respiratory signs and symptoms, Respiratory tract diseases

## Abstract

The Severe Acute Respiratory Syndrome Coronavirus 2 (SARS-CoV-2) Omicron variant associated myocardial injury seriously affected the patient's health. Chest computed tomography (CT) is an essential imaging diagnostic tool for evaluating lung diseases in these patients, but its value in the diagnosis of myocardial injury remains unknown. The purpose of this study was to evaluate the lung lesions in patients with Omicron infection with or without myocardial injury, and to evaluate the predictive value of non-contrast chest CT in such patients with myocardial injury. We enrolled 122 consecutive hospitalized patients with laboratory-confirmed coronavirus disease 2019 (COVID-19) for non-contrast chest CT examination. These patients were divided into two groups according to whether myocardial injury occurred. Myocardial injury was defined as a Troponin I level above the 99th-percentile upper reference limit (0.04 ng/mL). The imaging manifestations of the patients’ lungs were evaluated. Myocardial CT value, left atrium (LA) size, long diameter of left ventricular (LV), and cardiothoracic ratio (CTR) were recorded. Multivariate logistic analysis was performed to identify the predictive factors associated with myocardial injury. Of 122 patients, 61 patients (50%) had myocardial injury. Compared with patients without myocardial injury, there was worse NYHA class, more critical patients, higher incidence of bronchial meteorology, larger area and percentage of lung lesions, diameters of LA, and lower myocardial CT value in the myocardial injury group (P < 0.05). Troponin I concentration in patients with myocardial injury group showed negative correlation with myocardial CT value (r = − 0.319, P = 0.012). The multivariable logistic regression analysis showed that disease severity status (OR 2.279; 95% CI 1.247–4.165, *P* = 0.007), myocardial CT value (OR 0.849; 95% CI 0.752–0.958, *P* = 0.008), neutrophil count (OR 1.330; 95% CI 1.114–1.587, *P* = 0.002) were independent predictors of myocardial injury. The discrimination of the model was good (C-statistic = 0.845, 95% CI 0.775–0.914) and well calibrated with a Hosmer–Lemeshow test for goodness of fit (*P* = 0.476). Patients infected with Omicron with myocardial injury had more severe lung disease than those without myocardial injury. Non-contrast chest CT can be a useful method of detection of myocardial injury in Omicron infection patients.

## Introduction

For more than a year, emergence of the Severe Acute Respiratory Syndrome Coronavirus 2 (SARS-CoV-2) variant as Omicron has become a global concern^1^. The Omicron variant carries a variety of mutations that enhance its infectious and immune escape capacity, making it the dominant strain in many countries around the world. This poses new challenges for the prevention and control of coronavirus disease 2019 (COVID-19)^2^. Compared with the early original strains, the Omicron variant is the most mutated strain among the many SARS-CoV-2 variants arising during the COVID-19 pandemic^2^. The common clinical manifestations are: fever, cough, myalgia, fatigue, stuffy or runny nose, nausea, diarrhea, or loss of taste or smell^3^. SARS-CoV-2 not only invades the respiratory system but also causes other organ injuries in severe cases, such as cardiovascular diseases with an overall high prevalence and poor outcomes^4–9^. Furthermore, patients with known cardiovascular diseases are at an increased risk of developing a more severe form of COVID-19^10^. Therefore, increasing attention has been paid to cardiovascular adverse events caused by viral infection.

The detection and evaluation of myocardial injury are extremely important. In clinical practice, troponin elevation is often used as the standard to judge myocardial injury^11,12^. However, it is rare to utilize imaging tools to evaluate the cardiac function and histological characteristics of such myocardial injury. Patients infected with Omicron often cause lung diseases, and chest computed tomography (CT) is necessary to evaluate lung diseases. The soft tissue resolution of CT is relatively high, so the measurement of the myocardial CT value may play a certain auxiliary role in the judgment of myocardial injury while evaluating lung lesions.

The purpose of the present study is to evaluate the cardiac morphology and myocardial tissue characteristics while using non-contrast chest CT to evaluate lung lesions during the Omicron wave of the epidemic, thus increasing the clinical application value of chest CT in cardiovascular diseases and provide more prognostic information.

## Materials and methods

### Patients

We retrospectively enrolled 122 patients with laboratory confirmed COVID-19 Omicron who were admitted to the First Hospital of China Medical University from 1 December 2022 to 5 January 2023 during the Omicron wave of the pandemic and the general liberalization of societal control measures implemented as a result thereof. COVID-19 Omicron was diagnosed on the evidence of a positive result from real-time reverse transcriptase–polymerase chain reaction (RT-PCR) from clinical samples. The patients were divided into five levels: asymptomatic infected, mild, general, severe, and critical according to the Chinese management guideline for COVID-19 (Version 7). Patients with coronary heart diseases, cardiac pacemakers or other factors that affect the measurement of myocardial CT value were excluded. The current study was approved by the Ethics of Committees of The First Hospital of China Medical University. The patients provided their written informed consent to participate in this study. All methods were carried out in accordance with relevant guidelines and regulations.

### Chest CT acquisition

All patients were scanned in a supine position, arms up, head first, with a scanning range from the apex to the bottom of the lung, using GE 64 slice spiral CT scanning. GE 64 slice spiral CT parameters were as follows: scanning slice thickness 5 mm, reconstruction slice thickness 0.625 mm, reconstruction interval 0.625 mm; a high-resolution reconstruction algorithm was used to reconstruct the image, lung window settings (window width 1500 Hu, window level − 500 Hu) and mediastinal window setting (window width 350 Hu, window level 50 Hu) were adopted.

### Inter- and intra-observer reproducibility

Intra-observer reproducibility and inter-observer reproducibility were assessed by two radiologists with more than 2-year experience in 30 randomly selected patients (15 patients with myocardial injury and 15 patients without myocardial injury).

### Image analysis and post-processing

**Figure 1:**
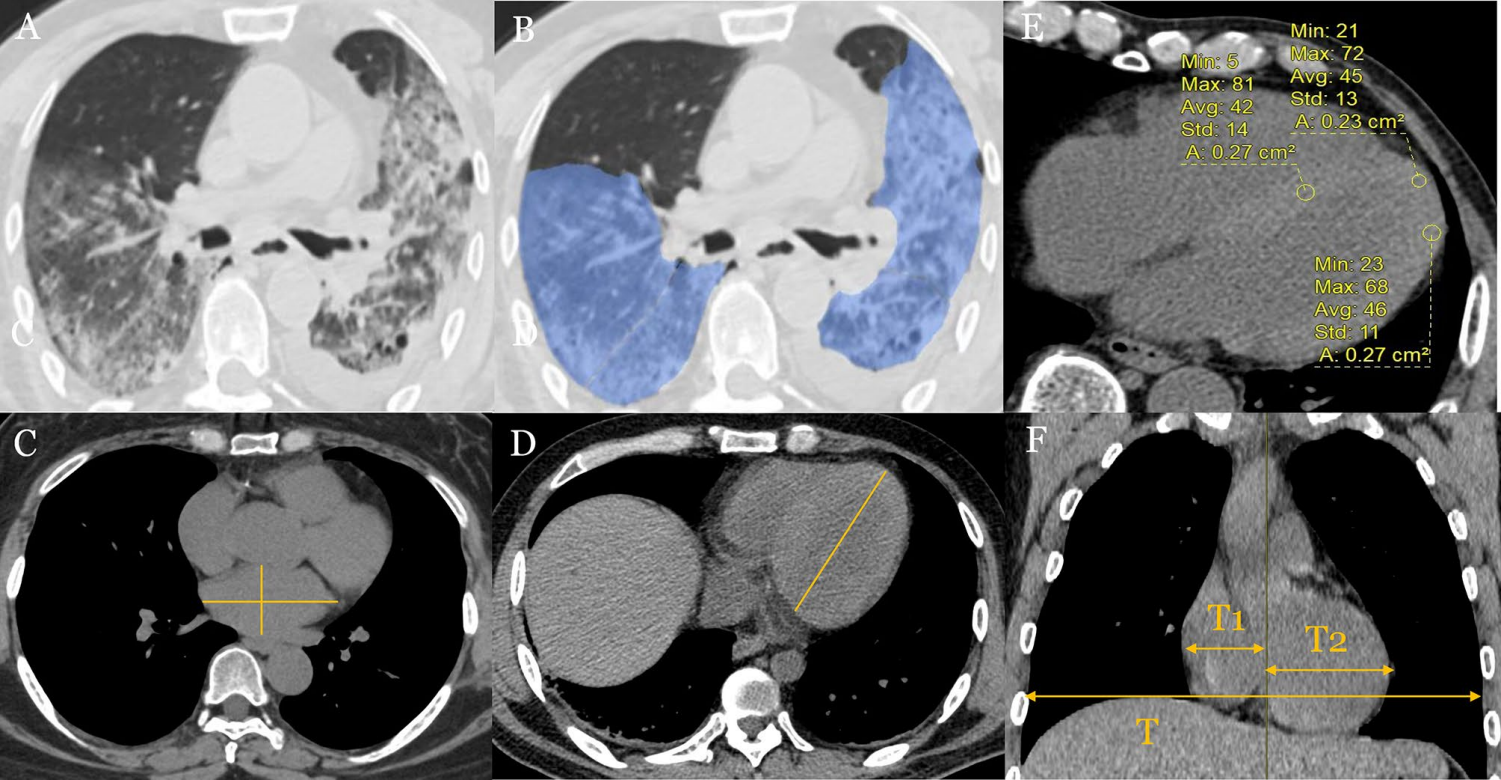
(**A**) A 74-year-old man with dyspnea, severe pneumonia. An axial CT image showed diffuse large regions of GGO, partial consolidation and pleural effusion. (**B**) Post-processing software showed the area and percent of GGO and consolidation. (**C**) Measurement of the anteroposterior diameter of LA and left and right diameters of LA. (**D**) Measurement of long diameter of LV. (**E**) Measurement of myocardial CT value. (**F**) Measurement of CTR (T1 + T2/T).

Several chest CT imaging features^13,14^ were mainly evaluated: (1) unilateral lung involvement or bilateral lung involvement; (2) ground glass opacity (GGO); (3) consolidation; (4) area and percentage of GGO or consolidation: intelligent diagnosis and treatment software for pulmonary diseases (Deepwise, Version 1.3.0.1, Hangzhou, China) (Fig. 1A,B); (5) air bronchogram; (6) bronchitis; (7) crazy-paving pattern; (8) reticulation; (9) left and right diameters and anteroposterior diameters of left atrium (LA): the anteroposterior diameter and the left and right diameters of the LA were measured at the maximum cross-section of the left atrium in the axial view of the mediastinal window of chest CT (Fig. 1C); (10) long diameter of left ventricular (LV): length from LV base to apex (Fig. 1D); (11) the myocardial CT value: the median axial slice first (slice 2) was selected, and then the slices 4–10 mm above the median axial slice (slice 1) and 4–10 mm below the median axial slice (slice 3) were selected. Three regions of interest (ROIs) were drawn in these slices: the middle of the interventricular septum, the anterior wall of LV and the lateral wall of LV. The ROI should be round^15^. During the measurement process, based on different myocardial thicknesses, the ROI area is approximately 0.2–0.3 cm^2^. The mean myocardial CT value of all ROIs was obtained (Fig. 1E); (12) cardiothoracic ratio (CTR): ratio of the maximum transverse diameter of the cardioid to the maximum transverse diameter of the thorax (*T*_1_ + *T*_2_/*T*) (Fig. 1F); (13) lymph node enlargement (hilum or mediastinum); (14) pleural effusion or pericardial effusion.

### Statistical analysis

SPSS 26.0 (IBM, Armonk, NY, USA) statistical software was used for statistical analysis. According to whether the statistics meet the normal distribution, the independent sample *t*-test or Wilcoxon rank sum test was selected for comparison among independent samples; continuous variables are expressed as mean ± standard deviation/median range. The classification variables were compared by *χ*^2^ test. Correlation analysis was performed to assess the association between Troponin I concentration in patients with the myocardial injury group and myocardial CT value. To explore potential determinants of myocardial injury, univariable logistic regression analysis of demographic, clinical and chest CT variables were performed; odds ratios were calculated with 95% confidence intervals (CI) as an estimate of the risk associated with each variable. Independent predictors were obtained by multivariable logistic regression analysis. Calibration of the final model was based on the Hosmer and Lemeshow test and discrimination on the *C*-statistic. Inter- and intra-observer reproducibility analyses were conducted by intraclass correlation coefficients (ICCs). An ICC of > 0.75 was considered to indicate high consistency. A two-tailed *P*-value of less than 0.05 was deemed statistically significant.

## Results

### Patient characteristics

We included a total of 122 patients. The patients were divided into two groups matched by sex and age according to whether myocardial injury occurred. Myocardial injury was defined as troponin I above the 99th-percentile upper reference limit (0.04 ng/mL). Table 1 summarizes the clinical characteristics in patients with and without myocardial injury. As shown in the Table 1, compared with patients without myocardial injury, there was a higher proportion of worse NYHA class, more critically ill patients in the myocardial injury group (*P* < 0.05). There was no significant difference in diabetes, smoking hypertension, ECG abnormalities and in-hospital death between patients with and without myocardial injury (*P* > 0.05).

**Table 1:** Comparisons of clinical characteristics between patients with and without myocardial injury. *NYHA* New York Heart Association, *ECG* electrocardiogram.

Variables	All (n = 122)	Patients with myocardial injury (n = 61)	Patients without myocardial injury (n = 61)	*P* value
Clinical characteristics				
Age, years	68 ± 15	73 (21)	68 ± 12	0.191
Male, n (%)	81 (66%)	42 (69%)	39 (64%)	0.565
Diabetes, n (%)	19 (16%)	9 (15%)	10 (16%)	0.803
Smoking, n (%)	9 (7%)	3 (5%)	6 (10%)	0.299
NYHA class ≥ II, n (%)	11 (9%)	10 (16%)	1 (2%)	**0.004**
Hypertension, n (%)	42 (34%)	23 (38%)	19 (31%)	0.446
ECG abnormalities	4 (3%)	3 (5%)	1 (2%)	0.309
Troponin I, ng/ml	0.042 (1.364)	1.331 (2.931)	0.004 (0.008)	**< 0.001**
In-hospital death, n (%)	6 (5%)	5 (8%)	1 (2%)	0.094
Disease severity status				
Mild, n (%)	5 (4%)	1 (2%)	4 (7%)	
General, n (%)	41 (34%)	19 (31%)	32 (52%)	**0.001**
Severe, n (%)	48 (39%)	25 (41%)	23 (38%)	
Critical, n (%)	18 (15%)	16 (26%)	2 (3%)	**0.001**

*P*-values of factors with bold values are less than 0.05.

### Comparisons of chest CT findings in patients with and without myocardial injury

In this study, COVID-19 infection often causes bilateral lung lesions in both groups. There was no difference in ground-glass opacities (GGO), consolidation, bronchitis, crazy-paving pattern, reticulation, lymph node enlargement, long diameter of LV, CTR > 0.5 and pericardial fusion (*P* > 0.05). But the area and percentage of GGO and consolidation, left and right diameters of LA, anteroposterior diameter of LA in the myocardial injury group were larger than those in the control group, the difference was statistically significant (*P* < 0.05). In the myocardial injury group, there were more signs of air bronchogram, pleural fusion than in the control group (*P* < 0.05). Myocardial CT value in the myocardial injury group was lower than that in the control group (44.42 ± 3.46 vs. 46.60 ± 4.05, *P* < 0.05). Detailed data are shown in Table 2.

**Table 2:** Comparisons of chest CT findings between patients with and without myocardial injury. *GGO* ground-glass opacities*, LA* left atrium, *LV* left ventricular, *CTR* cardiothoracic ratio.

Variables	All (n = 122)	Patients with myocardial injury (n = 61)	Patients without myocardial injury (n = 61)	*P* value
Bilateral, n (%)	109 (89%)	54 (89%)	55 (90%)	0.769
GGO, n (%)	90 (74%)	44 (72%)	46 (75%)	0.681
Consolidation, n (%)	88 (72%)	44 (72%)	44 (72%)	1.000
Area of GGO and consolidation, cm^3^	433.22 (890.52)	624.42 (1178.44)	393.08 (699.21)	**0.047**
Percent of GGO and consolidation, %	13.60 (26.58)	17.10 (40.20)	9.50 (20.95)	**0.029**
Air bronchogram, n (%)	54 (44%)	33 (54%)	21 (34%)	**0.029**
Bronchitis, n (%)	22 (18%)	12 (20%)	10 (16%)	0.638
Crazy-paving pattern, n (%)	10 (8%)	6 (10%)	4 (7%)	0.509
Reticulation, n (%)	40 (33%)	22 (36%)	18 (30%)	0.440
Lymph node enlargement, n (%)	47 (39%)	25 (41%)	22 (36%)	0.577
Left and right diameters of LA, cm	6.44 ± 1.15	6.65 ± 1.16	6.23 ± 1.10	**0.040**
Anteroposterior diameter of LA, cm	3.78 (0.97)	3.91 (1.53)	3.67 ± 0.67	**0.028**
Long diameter of LV, cm	8.10 ± 1.00	8.19 ± 1.25	8.01 ± 0.67	0.329
CTR > 0.5, n (%)	78 (64%)	42 (69%)	36 (59%)	0.258
Myocardial CT value, HU	45.51 ± 3.91	44.42 ± 3.46	46.60 ± 4.05	**0.002**
Pleural effusion, n (%)	26 (21%)	18 (30%)	8 (13%)	**0.027**
Pericardial effusion, n (%)	22 (18%)	15 (25%)	7 (11%)	0.060

*P*-values of factors with bold values are less than 0.05.

### Comparisons of laboratory findings in patients with and without myocardial injury

As shown in Table 3, white blood cell count, neutrophil count and procalcitonin in myocardial injury group were higher than those in the control group, lymphocyte count and eosinophil count were lower than those in the control group (*P* < 0.05). There was no difference in monocyte count, basophil count and C-reactive protein (CRP) in the myocardial injury group compared with the control group. Detailed data are shown in Table 3.

**Table 3:** Comparisons of laboratory findings between patients with and without myocardial injury.

Variables	All (n = 122)	Patients with myocardial injury (n = 61)	Patients without myocardial injury (n = 61)	*P* value
White blood cell count, × 10^9^/L	6.78 (4.69)	8.40 (6.25)	5.85 (2.88)	**< 0.001**
Neutrophil count, × 10^9^/L	4.98 (4.13)	6.44 (5.89)	4.09 (2.56)	**< 0.001**
Lymphocyte count, × 10^9^/L	0.93 (0.89)	0.76 (0.74)	1.22 (0.82)	**0.001**
Monocyte count, × 10^9^/L	0.48 (0.33)	0.42 (0.33)	0.51 (0.31)	0.099
Eosinophil count, × 10^9^/L	0.02 (0.05)	0.01 (0.04)	0.02 (0.05)	**0.031**
Basophil count, × 10^9^/L	0.01 (0.01)	0.02 (0.02)	0.01 (0.01)	0.152
C-reactive protein, mg/L	31.55 (68.43)	36.80 (94.22)	28.30 (38.30)	0.114
Procalcitonin, ng/mL	0.11 (0.48)	0.44 (2.13)	0.06 (0.08)	**< 0.001**

*P*-values of factors with bold values are less than 0.05.

### Correlation of myocardial CT value with troponin I

**Figure 2:**
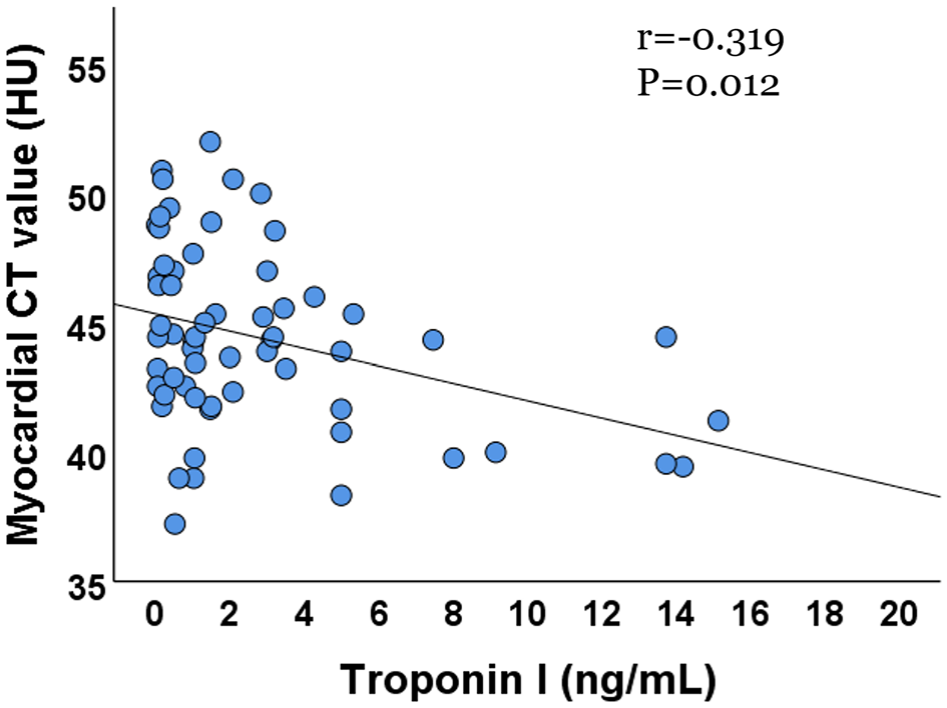
Correlation between Troponin I and myocardial CT value.

Correlation analysis showed that myocardial CT value was correlated with troponin I negatively (r = − 0.319, P = 0.012, Fig. 2).

### Independent determinants of myocardial injury

**Figure 3:**
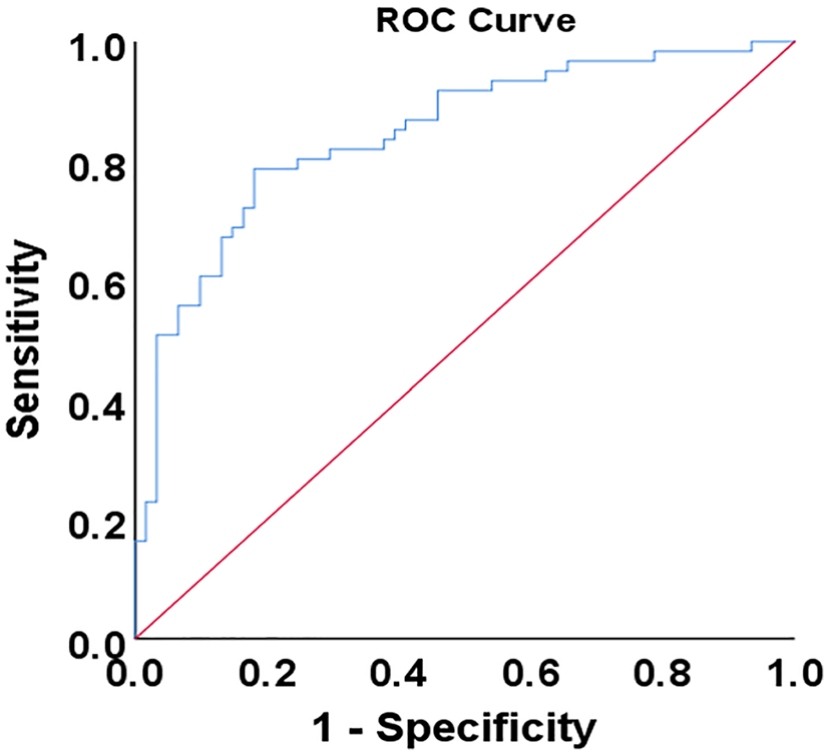
C-statistic of the multivariable logistic regression analysis model.

Univariable logistic regression analysis of clinical and CT features for myocardial injury is shown in the Table 4. As shown in Table 5, the multivariable logistic regression analysis showed that disease severity status (OR 2.279; 95% CI 1.247–4.165, *P* = 0.007), myocardial CT value (OR 0.849; 95% CI 0.752–0.958, *P* = 0.008) and neutrophil count (OR 1.330; 95% CI 1.114–1.587, *P* = 0.002) were independent predictors of myocardial injury. The discrimination of the model was good (C-statistic = 0.845, 95% CI 0.775–0.914) (Fig. 3) and well calibrated with a Hosmer–Lemeshow test for goodness of fit (*P* = 0.476).

**Table 4:** Univariable logistic regression analysis of clinical and CT features for myocardial injury. *OR* odds ratio, *CI* confidence interval*.*

Variable	Univariable analysis	
	OR (95% CI)	*P* value
Age, y	1.006 (0.983–1.029)	0.639
Male	1.247 (0.587–2.647)	0.566
Diabetes	0.883 (0.331–2.352)	0.803
Smoking	0.474 (0.113–1.990)	0.308
NYHA class ≥ II	11.765 (1.456–95.045)	**0.021**
Hypertension	1.338 (0.632–2.831)	0.446
ECG abnormalities	3.103 (0.314–30.701)	0.333
Disease severity status	2.699 (1.584–4.599)	**< 0.001**
GGO	0.844 (0.376–1.893)	0.681
Consolidation	1.446 (0.679–3.078)	0.339
Air bronchogram	2.245 (1.082–4.657)	**0.030**
Bronchitis	1.249 (0.495–3.154)	0.638
Crazy-paving pattern	1.555 (0.461–5.809)	0.512
Reticulation	1.348 (0.631–2.878)	0.441
Lymph node enlargement	1.231 (0.593–2.556)	0.557
Area of GGO and consolidation	1.001 (1.000–1.001)	**0.009**
Percent of GGO and consolidation	1.027 (1.007–1.048)	**0.007**
Left and right diameters of LA, cm	1.403 (1.010–1.948)	**0.043**
Anteroposterior diameter of LA, cm	1.913 (1.216–3.009)	**0.005**
Long diameter of LV, cm	1.199 (0.834–1.724)	0.328
CTR > 0.5	1.535 (0.729–3.231)	0.259
Myocardial CT value, HU	0.856 (0.772–0.948)	**0.003**
Pleural effusion	2.773 (1.100–6.993)	**0.031**
Pericardial effusion	2.516 (0.945–6.700)	0.065
White blood cell count, × 10^9^/L	1.231 (1.098–1.381)	**< 0.001**
Neutrophil count, × 10^9^/L	1.321 (1.321–1.532)	**< 0.001**
Lymphocyte count, × 10^9^/L	0.594 (0.344–1.025)	0.061
Monocyte count, × 10^9^/L	1.129 (0.853–1.493)	0.397
C-reactive protein, mg/L	1.010 (1.002–1.017)	**0.010**
Procalcitonin, ng/mL	1.474 (1.035–2.098)	**0.031**

*P*-values of factors with bold values are less than 0.05.

**Table 5:** Multivariable logistic regression analysis of clinical and CT features for myocardial injury.

Variable	Multivariable analysis	
	OR (95% CI)	*P* value
NYHA class ≥ II	7.706 (0.884–67.195)	0.065
Disease severity status	2.279 (1.247–4.165)	**0.007**
Air bronchogram		
Area of GGO and consolidation, cm^3^		
Percent of GGO and consolidation, %		
Left and right diameters of LA, cm		
Anteroposterior diameter of LA, cm		
Myocardial CT value, HU	0.849 (0.752–0.958)	**0.008**
Pleural effusion		
White blood cell count, × 10^9^/L		
Neutrophil count, × 10^9^/L	1.330 (1.114–1.587)	**0.002**
C-reactive protein, mg/L		
Procalcitonin, ng/mL		

*P*-values of factors with bold values are less than 0.05.

### Inter- and Intra-observer reproducibility

As shown in the Table 6, myocardial CT value exhibited good reproducibility.

**Table 6:** Intra- and inter-observer reproducibility for myocardial CT value. *ICC* intraclass correlation coefficient.

	Intra-observer	Inter-observer
	ICC (95% CI)	ICC (95% CI)
Myocardial CT value, HU	0.872 (0.749–0.937)	0.829 (0.671–0.915)

## Discussion

The purpose of this study was to evaluate the value of lung CT in detecting myocardial injury in patients with the Omicron-variant infection. It could be concluded that: (1) the myocardial CT value in the myocardial injury group was lower than that in the control group; (2) the myocardial CT value was negatively correlated with Troponin I; (3) disease severity status, myocardial CT value, and neutrophil count were independent predictors of myocardial injury.

COVID-19 infection is a systemic disease, which can affect all organs of the body. The incidence rate of myocardial injury caused by COVID-19 is high. In this study, Troponin I was used as the standard to judge whether there was myocardial injury. Patients infected by the Omicron variant were older and had certain underlying comorbidities, although there was no significant difference between the two groups, however, the NYHA in the myocardial injury group was worse than that in the control group. This is akin to the population with other variants of COVID-19^16,17^.

The respiratory tract is the first site of novel coronavirus infection. As the disease progresses, the patient may develop pneumonia. In this study, the proportion of GGO and consolidation in the lungs of the two groups was large, although there was no significant difference between the two groups. The clinical impact of pneumonia was found to be related to the increased risk of cardiovascular disease^18^. The percentage and area of GGO and/or consolidation in the myocardial injury group were larger than those in the group without myocardial injury. In addition, critical patients in the myocardial injury group were more numerous than those in the control group, while general patients in the non-myocardial injury group were more numerous than those in the myocardial injury group, suggesting that the more severe the Omicron-variant infection, the greater the myocardial injury, akin to other findings^5,19^. Moreover, our study found that disease severity status is one of the factors affecting myocardial injury, therefore, for patients with lung diseases, it is necessary to pay attention to myocardial injury.

The Omicron-variant infection caused a higher level of systemic inflammatory response, which was more evident in the myocardial injury group. In this study, the white blood cell count, neutrophil count, and procalcitonin in the myocardial injury group were higher than those in the non-myocardial injury group. Systemic inflammation caused by local infection of SARS-CoV-2 is believed to cause multiple organ damage including that to the heart^12,20^. Patients with the Omicron-variant infection may have different kinds of cardiovascular manifestations, including myocardial infarction, myocarditis, etc.^10,21,22^. Based on the aforementioned clinical manifestations, myocardial tissue manifestations may have different characteristics. Histological studies showed that myocardial interstitial edema and inflammatory cell infiltration could occur in the myocardium^22^. Myocardial injury caused by other strains of COVID-19 is associated with high mortality^7,9^. Therefore, the detection of myocardial injury in such patients is extremely important. Previous studies have shown that cardiac magnetic resonance found that the *T*_2_ value of myocardium in these patients are elevated; echocardiography also found abnormal cardiac function in these patients^22,23^. In this research, cardiac morphology and myocardial tissue were evaluated using chest CT. The anteroposterior diameter of LA and left and right diameters of LA of the myocardial injury group were higher than those of control group. The finding implied that this group of patients also had abnormal heart structure or that patients with abnormal heart structure were more likely to have myocardial injury. The myocardial CT value in myocardial injury group was lower than that of the control group, and the myocardial CT value was negatively correlated with Troponin I. The decrease of the myocardial CT value may indicate myocardial edema or myocarditis. In addition, the myocardial CT value is one of the predictors of myocardial injury. This suggests that Omicron-variant infection may cause cardiac abnormalities; myocardial CT value has a certain diagnostic value for myocardial injury. Based on this, chest CT has a certain diagnostic value in the detection of myocardial tissue and cardiac structural abnormalities in patients infected with the Omicron variant.

Omicron has strong infectivity and low toxicity, and the patient’s condition is relatively mild^21,24,25^. Research shows that among the hospitalized survivors of community-acquired pneumonia, systemic inflammation and procoagulant activity will continue to increase long after the indicator infection disappears^5,18^. In addition, some drugs used in clinical treatment of such patients may also lead to adverse cardiovascular events^26^. Therefore, although the upsurge of the Omicron-variant wave of the pandemic has gradually subsided, the long-term follow-up and management of the survivors of the Omicron-variant infection is crucial. Attention should be paid to the extrapulmonary manifestations and long-term consequences of patients with viral infection, and the interdisciplinary management of severe cases is essential. This study also proved the application value of chest CT in this disease.

### Limitations

This study has some limitations. It is a single-center study with a small sample size. There may be some deviation in the measurement of relevant cardiac parameters using non-contrast non-electrocardiogram (ECG)-gated chest CT, although the reproducibility of myocardial CT value was good. In future studies, we will focus on comparing the value of ECG-gated and non-ECG-gated CT imaging in assessing myocardial injury in such patients, in order to further confirm the value of non-ECG-gated CT imaging in evaluating myocardial injury. COVID-19 has produced several variants since the epidemic. Patients assessed in this study are all infected with the Omicron variant. Due to the limited collection sources of patients, it is impossible to obtain the data pertaining to the chest CT examination of patients infected with other viral variants, so it is impossible to compare the differences in data between patients infected with different viral variants. Because the patients were assessed within a short time, no follow-up and prognostic analysis was conducted for the patients in this study to evaluate the prognostic role of myocardial CT value for such patients. Cardiac dynamic function cannot be assessed.

## Conclusion

In conclusion, it was shown that reduced myocardial CT value indicated the occurrence of myocardial injury. Evaluation of lung lesions in patients with chest CT was conducted to check whether there was myocardial injury in such patients, thus making full use of imaging data to guide the early diagnosis of myocardial injury in clinical practice. Early treatment can improve the prognosis of patients, establish the correlation between imaging and clinical, and increase the clinical application value of chest CT.

## Data Availability

The datasets generated during and/or analyzed during the current study are available from the corresponding author on reasonable request.
